# Targeted demethylation of cathepsin D via epigenome editing rescues pathology in Alzheimer's disease mouse model

**DOI:** 10.7150/thno.103455

**Published:** 2025-01-01

**Authors:** Moonsu Park, Hongji Ryu, Suyeon Heo, Boyoung Kim, Junhang Park, Key-Hwan Lim, Sang-Bae Han, Hanseul Park

**Affiliations:** 1 Laboratory of Molecular Genetics, College of Pharmacy, Chungbuk National University, Cheongju, 28160, Republic of Korea.; 2 College of Pharmacy, Chungbuk National University, Cheongju, 28160, Republic of Korea.

**Keywords:** Alzheimer's disease, cathepsin D, epigenome editing, CRISPR/dCas9-Tet1, *in vivo* gene editing

## Abstract

**Background:** Cathepsin D (Ctsd) has emerged as a promising therapeutic target for Alzheimer's disease (AD) due to its role in degrading intracellular amyloid beta (Aβ). Enhancing Ctsd activity could reduce Aβ42 accumulation and restore the Aβ42/40 ratio, offering a potential AD treatment strategy.

**Methods:** This study explored Ctsd demethylation in AD mouse models using dCas9-Tet1-mediated epigenome editing. We identified dCas9-Tet1 as an effective tool for demethylating the endogenous Ctsd gene in primary neurons and *in vivo* brains.

**Results:** Treatment with Ctsd-targeted dCas9-Tet1 in primary neurons overexpressing mutant APP (mutAPP) reduced Aβ peptide levels and the Aβ42/40 ratio. Additionally, *in vivo* demethylation of Ctsd via dCas9-Tet1 in 5xFAD mice significantly altered Aβ levels and alleviated cognitive and behavioral deficits.

**Conclusion:** These findings offer valuable insights into developing epigenome editing-based gene therapy strategies for AD.

## Introduction

Alzheimer's disease (AD) is a progressive neurodegenerative disorder marked by memory loss and behavioral changes that severely impact social functioning 1-4. Pathologically, AD is characterized by the abnormal accumulation of two hallmark proteins: Aβ plaques and neurofibrillary tangles (NFTs) composed of hyperphosphorylated tau protein in the brain 5-7. These aggregates cause neuronal dysfunction, including axonal instability, impaired intracellular transport, disrupted synaptic communication, and ultimately neuronal death 7-9. Current treatments for AD are limited to symptomatic therapies that address neurotransmitter imbalances 10-12. However, no disease-modifying drugs capable of halting or significantly altering the disease's progression have been established 13, 14. Research continues to focus on developing treatments targeting the underlying pathogenic mechanisms of AD, such as the formation and deposition of extracellular Aβ plaques, intracellular NFTs, and related neuroinflammatory processes 5, 15, 16.

Cathepsin D (Ctsd) has emerged as a promising therapeutic target in AD 17. Ctsd, a soluble lysosomal aspartic endopeptidase encoded by the Ctsd gene, is synthesized in the rough endoplasmic reticulum as pre-procathepsin D (pre-proCTSD) 17. Ctsd functions as an intracellular aspartyl protease involved in degrading amyloid beta (Aβ) peptides. Research suggests that Ctsd levels increase in response to elevated Aβ expression in AD, indicating a compensatory mechanism aimed at mitigating Aβ accumulation 18, 19. Furthermore, a common genetic variation in the Ctsd gene has been associated with an increased risk for late-onset AD and elevated levels of both Aβ42 and tau proteins in cerebrospinal fluid 20. Interestingly, the genetic deletion of Ctsd significantly increases insoluble cerebral Aβ42, more so than the deletion of other Aβ-degrading enzymes like neprilysin 21. Also, Ctsd knockout (KO) mice show approximately 30% elevations in Aβ42/40 ratios, similar to changes induced by presenilin mutations 21. However, as AD progresses, Ctsd dysfunction appears to occur with the build-up of vacuolar structures and the accumulation of Aβ 20, 22. These findings emphasize Ctsd's crucial role in Aβ metabolism, suggesting that enhancing its expression may reduce Aβ accumulation and protect neurons from degeneration.

Precise downregulation of CTSD gene expression can be achieved through systems like shRNA and TRE-lox system 23, 24. However, to enhance CTSD gene expression for therapeutic purposes, current gene therapy methods primarily use cDNA-based overexpression 25-27. However, this approach often fails to precisely regulate endogenous gene activity, as cDNA is typically introduced under a strong exogenous promoter, leading to high expression levels independent of the gene's native regulatory elements 28-31. This can exceed physiological protein levels without restoring the natural gene expression pattern necessary for cellular homeostasis. Catalytically inactivated Cas9 (dCas9)-based epigenome editing offers a promising alternative for regulating endogenous genes. dCas9, an endonuclease-deficient variant of the clustered regularly interspaced short palindromic repeats (CRISPR)/Cas9 system, is a versatile tool for targeted gene modification 32-34. When combined with specific effector domains, dCas9 can be used for various genomic engineering applications, including gene activation/repression, histone acetylation/deacetylation, and DNA methylation/demethylation 35-37. For instance, fusing dCas9 with Ten-eleven translocation 1 (Tet1) enables precise DNA demethylation of the target gene 38-40. This dCas9-Tet1-mediated demethylation can effectively target CpG islands (CGIs), modulating gene expression with high specificity 41-43. In our recent study, we demonstrated that targeted methylation of the amyloid precursor protein (APP) gene using dCas9-Dnmt3a could be a potential therapeutic strategy for AD 44. This finding underscores DNA demethylation editing as an innovative approach for precisely regulating gene expression in treating various diseases.

In this study, we used a dCas9-Tet1-mediated DNA demethylation system to induce Ctsd hypomethylation, aiming to upregulate Ctsd expression in primary neurons and the mouse brain *in vivo*. First, we validated that dCas9-Tet1 effectively demethylated the Ctsd promoter region, successfully increasing Ctsd expression in the mouse brain. We then evaluated Aβ42 formation and Aβ-associated memory impairment in the 5xFAD mouse model to assess the therapeutic potential of Ctsd-targeted demethylation as a treatment strategy for AD.

## Methods

### Production of guide RNAs

The pLenti-sgRNA vector (Addgene, #71409) was utilized to clone a single guide RNA (sgRNA). To prepare the vectors, they were linearized using the BsmbI enzyme (enzynomics) at 56°C for 2 h. Following linearization, sgRNA oligonucleotide pairs were annealed with T4 polynucleotide kinase (NEB) and subsequently ligated into the linearized vectors (50 ng) using T4 DNA ligase (NEB) at room temperature for 2 h. The ligation product was then transformed into DH5α competent E. coli cells (enzynomics). Successful ligation was verified by restriction enzyme digestion. Additionally, the plasmids were sequenced using the primer GAGGGCCTATTTCCCATGATT to confirm the correct insertion of the sgRNA sequences.

### Animal experiments

All animal experiments were conducted in accordance with the guidelines set by the Institutional Animal Care and Use Committee of Chungbuk National University. The mice were housed in a controlled environment with a 12-h light/dark cycle and a stable temperature of 22°C-23°C. The study utilized 5xFAD transgenic mice, which co-express five familial Alzheimer's disease mutations: APP K670N/M671L (Swedish, double mutation), V717I (London), I716V (Florida), and PS1 with two FAD mutations, M146L and L286V. These mice were obtained from The Jackson Laboratory (stock no. 006554) 45. Experiments were conducted on 5-month-old male 5xFAD and B6C57 mice. The mice were anesthetized with Avertin (2,2,2-tribromoethanol; Sigma) at a dose of 120 mg/kg. A total of 10 µl of either dCas9-Tet1, empty vector (control), or CTSD-336 sgRNA lentivirus was microinjected into the dentate gyrus (DG) of both hemispheres. The injection coordinates were anteroposterior (AP) -2 mm, mediolateral (ML) ±1.1 mm, and dorsoventral (DV) -2 mm. Behavioral tests included the Y-maze, fear conditioning, and water maze tests, with mouse activity recorded and analyzed using Noldus EthoVision XT 13 software (Noldus, the Netherlands). In the Y-maze test, mice were placed in a maze with three open arms, and their spontaneous alternation behavior was recorded for 10 min. The total number of arm entries was used to calculate visitation. Fear conditioning tests spanned 2 consecutive days. On day 1, mice explored a conditioning chamber for 3 min, followed by paired stimuli (1 s, 0.7 mA) and a subsequent minute to assess freezing behavior. On day 2, conditioned fear was evaluated by observing freezing behavior for 2 min. The water maze test was conducted in a circular tank, with mice trained for 4 days, three trials per day, during the visible platform test. On day 4, in the invisible platform test, the time spent in each quadrant was recorded for 1 min. After completing the behavioral tests, 6-month-old male mice were sacrificed for biochemical analysis.

### Cell culture

NIH/3T3, Neuro-2a (N2a), and mouse embryonic fibroblast (MEF) cells were cultured in Dulbecco's Modified Eagle Medium (Gibco) with heat-inactivated fetal bovine serum (FBS; Gibco) and 1% penicillin/streptomycin (P/S; Gibco). Primary neurons were harvested from 5xFAD mice at embryonic day 14 (E14) and cultured in Neurobasal medium (Gibco) supplemented with heat-inactivated FBS, B-27TM (Gibco, MA, USA), 200 mM L-glutamine (Gibco), P/S, and laminin (Corning). Neurons were maintained for 2 weeks before biochemical analysis. All cell cultures were kept at 37°C with 5% CO2. Cell line authentication was confirmed by Short Tandem Repeat profiling (Kogene Biotech), and mycoplasma contamination was checked using the MycoSensor PCR Assay Kit (Agilent). For lentivirus production, HEK293T cells were cultured to 80% confluence before transfection with lentiviral plasmids (psPAX2, pMD2.G, and either pLenti-sgRNA [Addgene, #71409] or Fuw-dCas9-Tet1CD [Addgene, #84475]). Transfection was performed using calcium phosphate (Sigma) and HEPES buffer (Sigma). After 20 h, the medium was replaced, and viral particles were collected by centrifugation 72 h post-transfection.

### Off-target analysis

Off-target sequences were predicted using the Cas-OFFinder tool (http://www.rgenome.net/cas-offinder). To validate these potential off-targets, the top six sites with two or fewer mismatches compared to the on-target sequence were analyzed using quantitative real-time PCR (qRT-PCR).

### Immunocytochemistry

Cells and hippocampal brain tissue were fixed in 4% paraformaldehyde (Sigma) and washed with phosphate-buffered saline (PBS). Following a 20-min block with PBST containing 1% BSA, samples were incubated overnight at 4°C with primary antibodies, including anti-NeuN (Invitrogen, PA5-78639), anti-Tuj1 (Sigma, T8578), anti-Map2 (Thermo Fisher Scientific, 13-1500), and anti-Aβ 1-42 (Abcam, ab201060). After washing with PBST, samples were incubated with secondary antibodies for 2 h at room temperature, then counterstained with 4′, 6-Diamidino-2-phenylindole dihydrochloride (DAPI) (Invitrogen). Visualization was performed using a Zeiss LSM 700 confocal microscope. The percentage of Aβ42+/Map2+ cells was calculated by determining the ratio of double-positive Aβ42 and Map2 cells to the total number of Map2-positive cells.

### Thioflavin T (ThT) staining

Brain sections were washed with 0.1 M Tris-buffered saline (pH 7.5) and stained with ThT (Merck, #596200) in 50% ethanol for 10 min. After rinsing in Tris-buffered saline, the sections were immunolabeled with an anti-Aβ antibody (1:250; rabbit polyclonal anti-beta amyloid, Abcam, #ab2539) and then treated with a fluorescence-conjugated secondary antibody. The experimenter was aware of the treatment groups, and all cell cultures were included in the analysis.

### RNA isolation and qRT-PCR

Total RNA was extracted using the eCube Tissue RNA Mini Kit (Philekorea) following the manufacturer's instructions. Next, 1 μg of RNA was reverse transcribed into cDNA using AccuPower® CycleScript RT PreMix (Bioneer). Quantitative real-time PCR (qRT-PCR) was conducted on the Rotor-Gene Q system (Qiagen) with specific primers and AccuPower® PCR PreMix (Bioneer) for amplification.

### Western blot analysis

Cell and brain tissue samples were lysed in RIPA buffer (Sigma) containing 1× protease inhibitor cocktail (Sigma) and 5× loading buffer. The lysates were heated to 100°C for 10 min, then centrifuged at 14,000 × g for 10 min to remove debris. Supernatants were separated by SDS-PAGE and transferred to membranes. Membranes were incubated overnight at 4°C with primary antibodies against cathepsin D (Santa Cruz Biotechnology, sc-377299) or β-actin (AbFrontier, LF-PA0207). After washing, membranes were treated with HRP-conjugated secondary antibodies, and protein bands were detected using an enhanced chemiluminescence (ECL) kit (Dogen).

### Bisulfite sequencing

Total DNA was isolated using the eCube Tissue DNA Mini Kit (Philekorea) following the manufacturer's instructions. Two micrograms of DNA were subjected to bisulfite conversion using the EpiTect Bisulfite Kit (Qiagen). PCR amplification of the bisulfite-treated DNA was carried out using custom primers from PrimerSuite (www.primer-suite.com). The amplified DNA fragments were purified with the NucleoSpin® Gel and PCR Clean-up Kit (Macherey-Nagel) and subsequently cloned into vectors using the TA Cloning™ Kit (Thermo Fisher Scientific) for sequencing analysis.

### Aβ42 and Aβ40 quantification

Cell and brain tissue samples were lysed in RIPA buffer (Sigma) with 1× protease inhibitor cocktail (Sigma). Aβ40 and Aβ42 concentrations were measured using Aβ (1-40) and Aβ (1-42) (FL) ELISA kits (IBL International). Absorbance was recorded at 450 nm using a VERSAmax tunable microplate reader (Molecular Devices).

### Statistical analysis

Statistical analysis was conducted with GraphPad Prism 9.3.1 software. Differences between groups were assessed using one-way and two-way ANOVA, as well as two-tailed unpaired t-tests. A *p* value of < 0.05 was considered statistically significant.

## Results

### Targeted DNA demethylation of the Ctsd promoter locus using dCas9-Tet1

**Figure 1:**
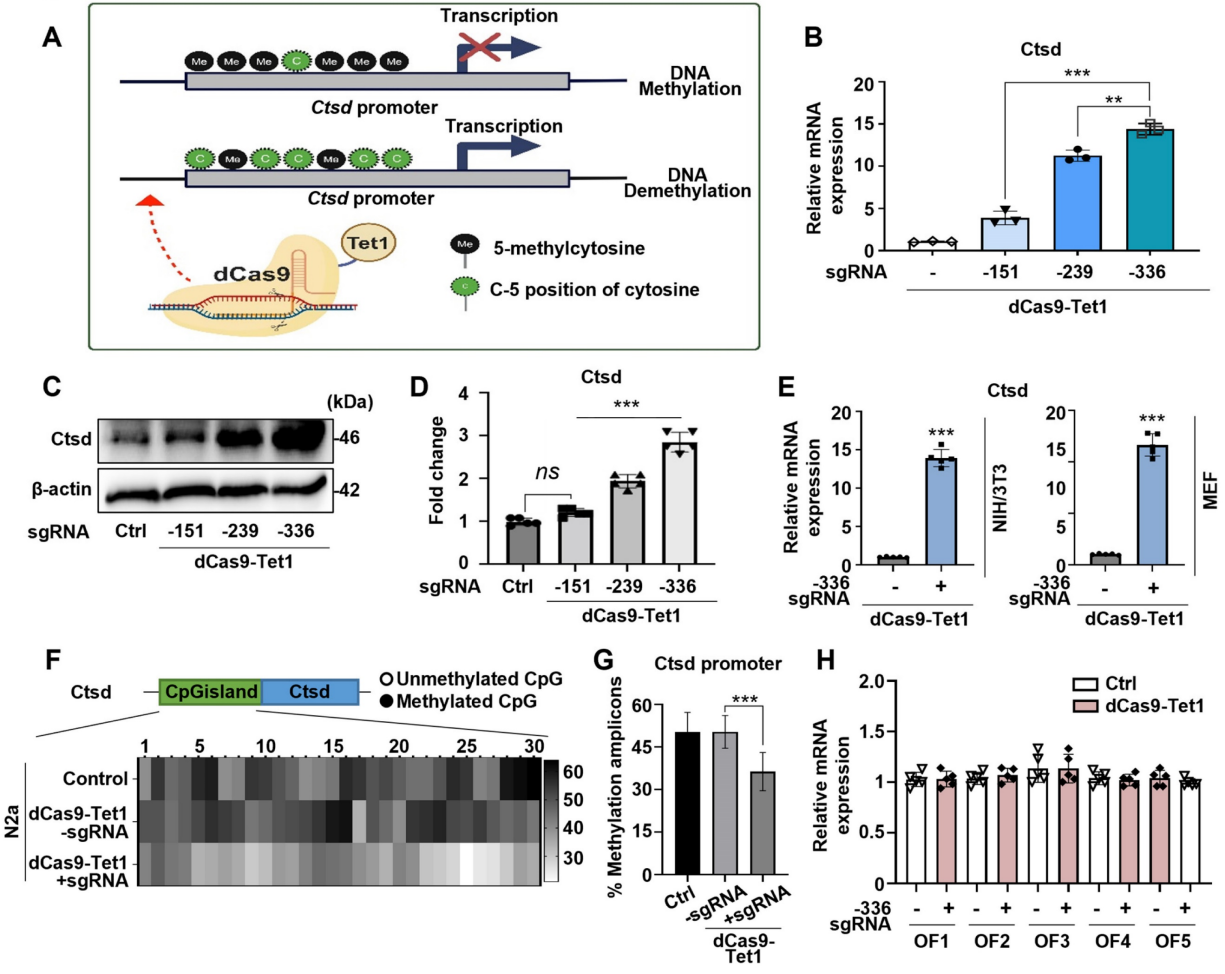
** Demethylation of Ctsd by dCas9-Tet1 *in vitro*.** (A) Schematic representation of the dCas9-Tet1-mediated demethylation system targeting the Ctsd promoter with specific sgRNAs. (B) Quantitative real-time PCR analysis of Ctsd mRNA expression in N2a cells with sgRNAs targeting positions -151, -239, and -336 bp upstream of the Ctsd start codon. Data are presented as mean ± SEM (*n* = 3). **p < 0.01, ***p < 0.001, one-way ANOVA with Tukey's multiple comparisons test. (C-D) Western blot analysis of Ctsd demethylation in N2a cells treated with sgRNAs targeting positions -151, -239, and -336. Data are expressed as mean ± SEM (*n* = 5). *p < 0.05, **p < 0.01, one-way ANOVA with Tukey's multiple comparisons test. (E) Quantitative real-time PCR analysis of Ctsd mRNA expression in NIH/3T3 and MEF cells with sgRNA targeting position -336. Data are presented as mean ± SEM (*n* = 5). ***p < 0.001, two-tailed unpaired t-tests. (F) Bisulfite sequencing analysis of the Ctsd promoter region in N2a cells treated with sgRNA targeting -336 and dCas9-Tet1. (G) Quantification of methylated amplicons across three independent experiments, analyzing 10 to 30 sequences per experiment. Data are derived from two biological replicates. ***p < 0.001, one-way ANOVA with Tukey's multiple comparisons test. (H) Evaluation of off-target effects using Cas-offinder. Data are presented as mean ± SEM (*n* = 5). *p < 0.05, two-tailed unpaired t-tests. Images in panels B-C represent data from three or more similar experiments.

Ctsd, a lysosomal protease involved in degrading Aβ and tau, is genetically linked to late-onset AD 46-48. Reduced Ctsd activity leads to Aβ accumulation and accelerates AD progression 49-51. The potential of the dCas9-Tet1 system to modulate DNA demethylation at Ctsd CGI regions was evaluated. Three single guide RNAs (sgRNAs) targeting Ctsd CGI regions, predicted to have the highest binding efficiency for DNA demethylation (Figure 1A and Table S1), were designed. The efficacy of these sgRNAs combined with dCas9-Tet1 was tested in N2a cells. Results showed a significant increase in Ctsd expression with Ctsd-specific sgRNAs (Figure 1A-B). The sgRNA targeting -336 bp upstream of the Ctsd start codon resulted in the most substantial Ctsd expression increase (Figure 1A-B). Additionally, the combination of -336 sgRNA and dCas9-Tet1 significantly elevated Ctsd protein levels in N2a cells (Figure 1C-D). Thus, the dCas9-Tet1 system with -336 sgRNA was chosen for further experiments on Ctsd DNA demethylation.

To further validate our findings, we confirmed the increased Ctsd expression in NIH/3T3 and MEF cell lines. The dCas9-Tet1 system combined with the -336 sgRNA significantly elevated Ctsd mRNA levels in both NIH/3T3 and MEF cell lines (Figure 1E). DNA demethylation within the Ctsd CGI region was confirmed by bisulfite sequencing, which revealed a marked increase in hypomethylation in N2a cells treated with the -336 sgRNA and dCas9-Tet1 (Figure 1F-G). To assess potential off-target effects, Cas-OFFinder, a web-based off-target prediction tool, was used to identify possible genome-wide off-target sites. Analysis of five predicted off-target sites in N2a cells showed no detectable alterations induced by the -336 sgRNA and dCas9-Tet1 (Figure 1H and Table S2). Collectively, these results demonstrate that the dCas9-Tet1 system effectively demethylates the Ctsd CGI region, enhancing Ctsd expression in cells without significant off-target effects.

### Demethylation of Ctsd decreases Aβ42/40 ratios in mutAPP mouse primary neurons

**Figure 2:**
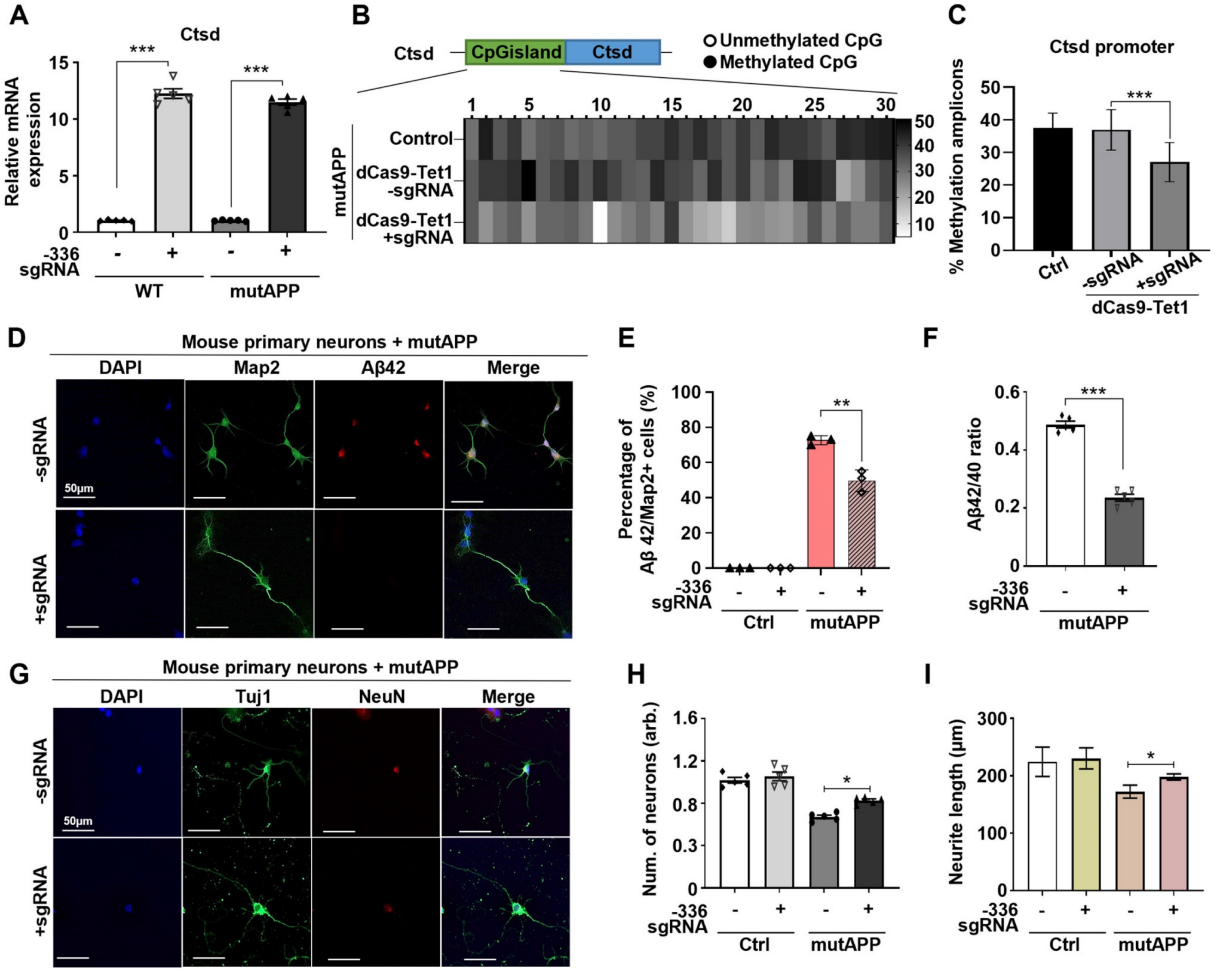
** dCas9-Tet1-mediated demethylation of Ctsd reduced Aβ42 production in mutAPP mouse primary neurons.** (A) Quantitative real-time PCR analysis of Ctsd expression in primary neurons from WT and mutAPP mice treated with -336 sgRNA and dCas9-Tet1. Data are shown as mean ± SEM (n = 5). *** p < 0.001, two-tailed unpaired t-tests. (B) Bisulfite sequencing analysis of the Ctsd promoter region in mutAPP mouse primary neurons treated with -336 sgRNA and dCas9-Tet1. (C) Quantification of demethylated amplicons. A total of 10 to 30 sequences were analyzed across three independent experiments. Data are expressed as mean ± SEM. *** p < 0.001, one-way ANOVA with Tukey's multiple comparisons test. (D) Immunostaining for Map2 (green), Aβ42 (red), and DAPI (blue) in mutAPP mouse primary neurons treated with -336 sgRNA and dCas9-Tet1. (E) Quantification of Aβ42+/Map2+ cell counts from panel D. More than 100 neurites were measured across three independent experiments. Data are presented as mean ± SEM (n = 5). **p < 0.01, two-way ANOVA with Tukey's multiple comparisons test. (F) ELISA assessment of the Aβ42/Aβ40 ratio in mutAPP mouse primary neurons transduced with -336 sgRNA and dCas9-Tet1. Data are expressed as mean ± SEM (n = 5). ***p < 0.0001, two-tailed unpaired t-tests. (G) Immunostaining for Tuj1 (green), NeuN (red), and DAPI (blue) in mutAPP mouse primary neurons treated with -336 sgRNA and dCas9-Tet1. (H) Quantification of Tuj1-positive neurons from panel G. Data are shown as mean ± SEM (n = 5). *p < 0.05, two-tailed unpaired t-tests. (I) Quantification of neurite length from panel G. More than 100 neurites were measured across three independent experiments. Data are expressed as mean ± SEM. *p < 0.05, two-tailed unpaired t-tests. Images in panels B, D, and G are representative of three or more similar experiments.

Ctsd is the primary intracellular protease identified to degrade Aβ42 21. To determine if dCas9-Tet1-mediated hypomethylation of Ctsd could reduce Aβ42 levels and provide neuroprotection, we tested this in mouse primary neurons overexpressing mutAPP *in vitro*. Transduction of the -336 sgRNA and dCas9-Tet1 into primary neurons, with or without mutAPP overexpression, resulted in a significant increase in Ctsd mRNA expression in both wild-type (WT) and mutAPP primary neurons (Figure 2A). Also, bisulfite sequencing analysis confirmed hypomethylation of the Ctsd CGI region in primary neurons treated with the -336 sgRNA and dCas9-Tet1, correlating with the observed upregulation of Ctsd expression (Figure 2B-C).

Given that deletion of Ctsd increases endogenous Aβ42 and Aβ40 levels 21, we investigated the impact of Ctsd hypomethylation via dCas9-Tet1 on Aβ levels in mutAPP primary neurons. Following transduction with the -336 sgRNA and dCas9-Tet1, Aβ42 levels were quantified in mutAPP primary neurons. Results showed a significant decrease in the number of Aβ42 and Map2 double-positive neurons in dCas9-Tet1-treated mutAPP primary neurons (Figure 2D-E). We also observed reduced levels of Aβ42 and Aβ40 in dCas9-Tet1 transduced primary neurons, with Aβ42 levels showing a more pronounced decrease than Aβ40 (Figure S1A-B). Thus, dCas9-Tet1 transduction effectively reduces both Aβ42 and the Aβ42/Aβ40 ratio in mutAPP primary neurons (Figure 2F, Figure S1A-B). Furthermore, we assessed dendritic spine density in mutAPP primary neurons after dCas9-Tet1 transduction. At 14 days *in vitro*, mutAPP primary neurons showed reduced neurite length of neuronal-specific genes (Tuj1 and NeuN) (Figure 2G-H). However, transduction with the -336 sgRNA and dCas9-Tet1 increased both neuron number and neurite length in mutAPP primary neurons (Figure 2G-I). These findings demonstrate that targeting Ctsd with dCas9-Tet1 enhances Ctsd hypomethylation and mitigates AD-associated phenotypes in mutAPP primary neurons.

### *In vivo* dCas9-Tet1 mediated Ctsd demethylation in mouse brain

**Figure 3:**
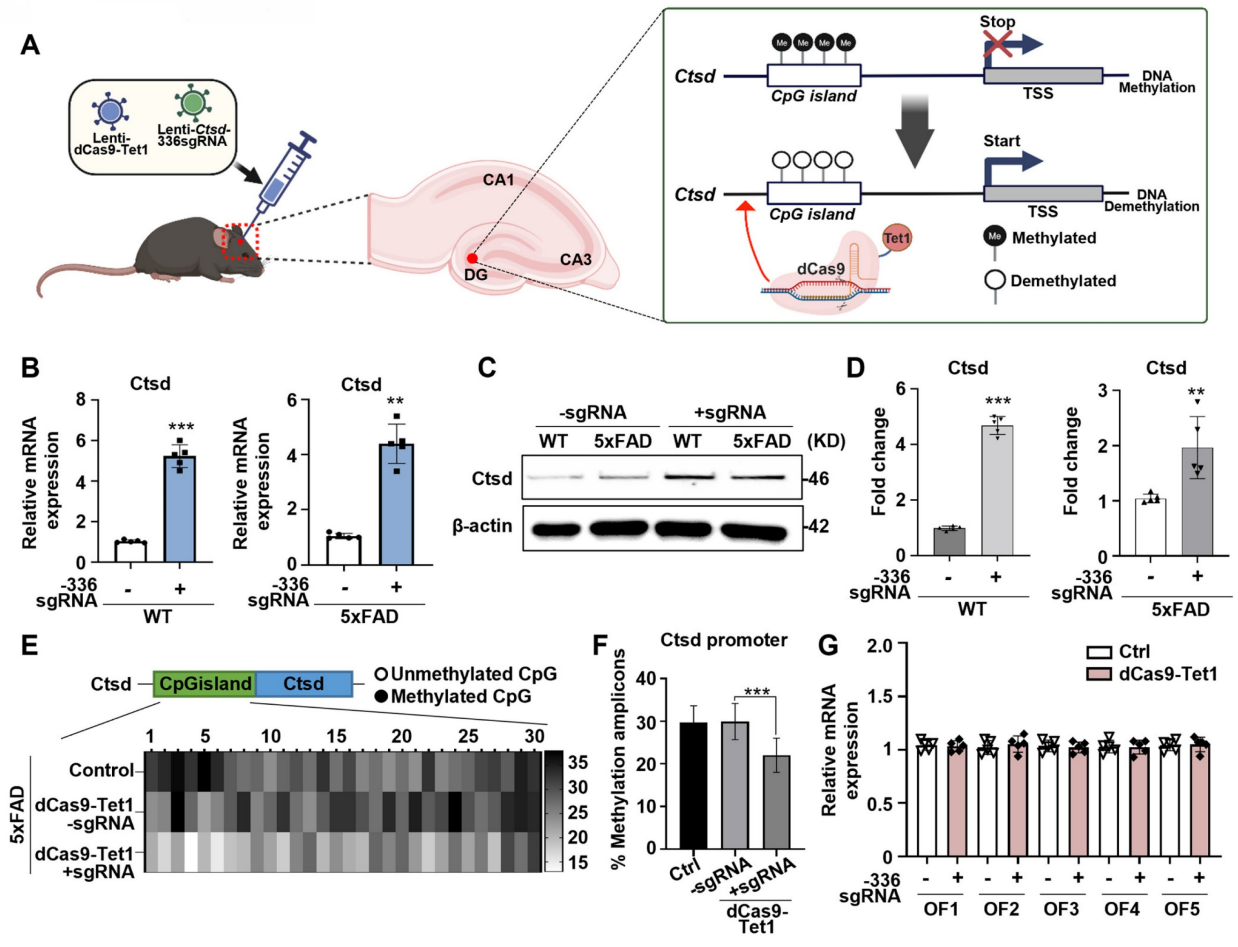
** dCas9-Tet1-Mediated demethylation of Ctsd in mouse brain.** (A) Schematic representation of the dCas9-Tet1-mediated demethylation system targeting the Ctsd promoter in the mouse brain *in vivo*. (B) Quantitative real-time PCR analysis of Ctsd expression in the brains of WT and 5xFAD mice injected with -336 sgRNA and dCas9-Tet1. Data are shown as mean ± SEM (n = 5). **p < 0.01, ***p < 0.001, two-tailed unpaired t-tests. (C-D) Western blot analysis of Ctsd in the brains of WT and 5xFAD mice injected with -336 sgRNA and dCas9-Tet1. Data are presented as mean ± SEM (*n* = 3). **p < 0.01, ***p < 0.001, two-tailed unpaired t-tests. (E) Bisulfite sequencing analysis of the Ctsd promoter region in the brain of 5xFAD mice injected with -336 sgRNA and dCas9-Tet1. (F) Quantificationof demethylated amplicons. A total of 10 to 30 sequences were analyzed across three independent experiments. Data are expressed as mean ± SEM. ***p < 0.001, one-way ANOVA with Tukey's multiple comparisons test. (G) Evaluation of off-target effects using Cas-offinder in the hippocampus of WT mice injected with -336 sgRNA and dCas9-Tet1. Data are presented as mean ± SEM (*n* = 5). *p < 0.05, two-tailed unpaired t-tests. Image in panel C is representative of three or more similar experiments.

To evaluate whether dCas9-Tet1-mediated demethylation could be applied for *in vivo* Ctsd demethylation, we injected lentivirus containing dCas9-Tet1 with Ctsd sgRNA directly into the DG of mouse brains (Figure 3A). Transduction with the -336 sgRNA and dCas9-Tet1 significantly increased Ctsd mRNA levels in the brains of WT and 5xFAD AD mice (Figure 3B). Western blot analysis confirmed that baseline Ctsd levels were higher in 5xFAD mice compared to WT mice, and that transduction with -336 sgRNA and dCas9-Tet1 significantly increased Ctsd protein levels in both WT and 5xFAD mice (Figure 3D). We also assessed whether dCas9-Tet1 could enhance Ctsd hypomethylation in the brain. Bisulfite sequencing revealed significant demethylation of the Ctsd locus CGIs following dCas9-Tet1 transduction compared to age-matched control mice (Figure 3E-F). To assess the persistence of gene activation following dCas9-Tet1 brain injection, we found that Ctsd expression levels remained elevated for up to 144 hours post-injection (Figure S2). This sustained activation demonstrated that the dCas9-Tet1 system can effectively regulate Ctsd expression *in vivo* brain. Also, to evaluate potential *in vivo* off-target effects, we analyzed predicted off-target sites (Figure 3G and Table S2). Consistent with *in vitro* findings (Figure 1H), the -336 Ctsd sgRNA did not show detectable off-target activity *in vivo* (Figure 3G). These results demonstrate that dCas9-Tet1 effectively targets Ctsd demethylation in the brain with high specificity, without causing off-target effects.

### Demethylation of Ctsd via dCas9-Tet1 reduces Aβ42 and improves cognition in the AD mouse model

**Figure 4:**
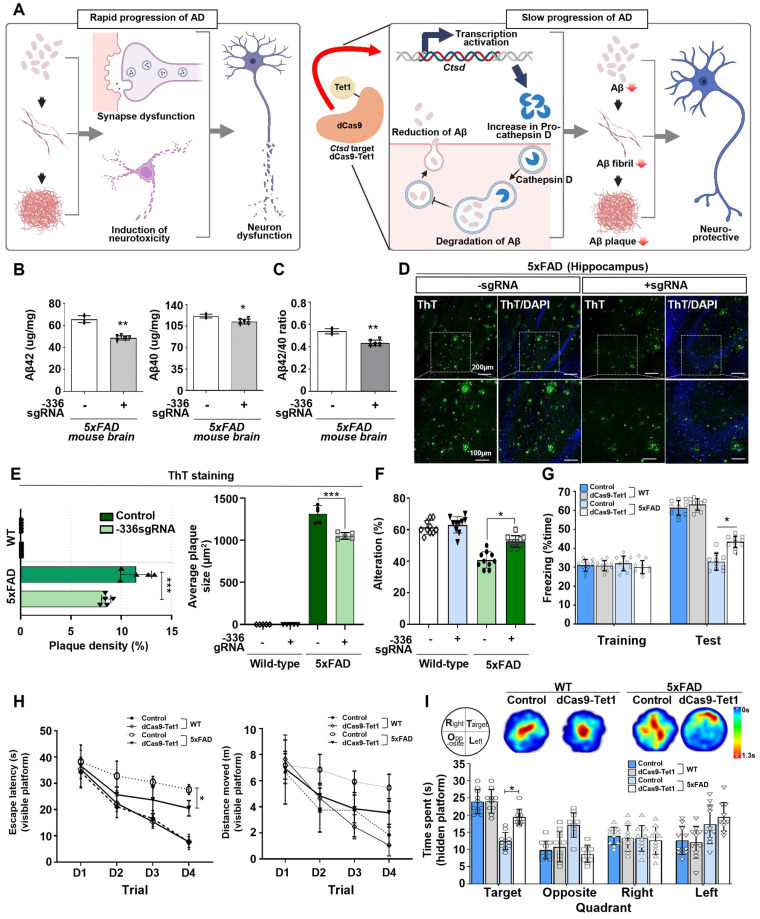
** dCas9-Tet1-Mediated Demethylation of Ctsd results in therapeutic effects in the 5xFAD mouse model.** (A) Schematic representation of the dCas9-Tet1-mediated demethylation system based on the 5xFAD mouse model. (B-C) ELISA analysis of Aβ42, Aβ40, and the Aβ42/Aβ40 ratio in the brains of 5xFAD mice injected with -336 sgRNA and dCas9-Tet1. Data are presented as mean ± SEM (n = 5). *p < 0.05, **p < 0.01, two-tailed unpaired t-tests. (D) ThT assay for amyloid aggregation. ThT-positive (green) and DAPI (blue) immunostaining in the brain of 5xFAD mice injected with -336 sgRNA and dCas9-Tet1. (E) Quantification of the Aβ42 region from panel D. Data are shown as mean ± SEM (*n* = 5). ***p < 0.001, two-way ANOVA with Tukey's multiple comparisons test. (F) Spontaneous alternation in the Y-maze for WT and 5xFAD mice injected with -336 sgRNA and dCas9-Tet1. Data are expressed as mean ± SEM. *p < 0.05, two-way ANOVA with Tukey's multiple comparisons test. (G) Freezing behavior analysis using the contextual fear memory test. Data are presented as mean ± SEM. *p < 0.05, two-way ANOVA with Tukey's multiple comparisons test. (H-I) Long-term spatial memory analysis using the Morris water maze test. (H) Visible platform training session analysis showing escape latency (left) and total distance moved (right). Data are expressed as mean ± SEM. *p < 0.05, two-way ANOVA with Tukey's multiple comparisons test. (I) Hidden platform test session analysis including heatmap and quantification of quadrant occupancy time. Data are shown as mean ± SEM. *p < 0.05, two-way ANOVA with Tukey's multiple comparisons test. Image in panel D is representative of three or more similar experiments.

AD is characterized by the accumulation of abnormal amounts of Aβ, which forms amyloid plaques outside neurons and tau proteins, leading to neurofibrillary tangles inside cells. These abnormalities progressively impair neural function and connectivity, resulting in a gradual decline in brain function 52-54. Given that enhancing Ctsd activity could potentially reduce Aβ42 levels 21, we investigated whether demethylation of Ctsd via dCas9-Tet1 in the mouse brain could decrease Aβ42 levels and alleviate cognitive and memory impairments in the 5xFAD mouse model. We injected dCas9-Tet1 and -336 sgRNA into the DG of the 5xFAD mouse brain and conducted biochemical and behavioral analyses 4 weeks post-injection (Figure 4A). To evaluate the effect of Ctsd demethylation on Aβ pathology, we measured Aβ42 and Aβ40 levels in the 5xFAD mice. Four weeks after injection, dCas9-Tet1 treatment resulted in a significant reduction in both Aβ42 and Aβ40 levels compared to age-matched 5xFAD controls (Figure 4B). The Aβ42/Aβ40 ratio in the hippocampus of 5xFAD mice also decreased significantly following dCas9-Tet1 treatment (Figure 4C). Additionally, Aβ42 plaque accumulation in the hippocampus was markedly reduced by dCas9-Tet1 (Figure 4D-E). These findings indicate that dCas9-Tet1-mediated Ctsd demethylation effectively reduced Aβ levels in 5xFAD mice.

Subsequently, behavioral assays were conducted to evaluate the impact of Ctsd demethylation via dCas9-Tet1 on cognitive functions. We used both short-term and long-term memory tests. The Y-maze test assessed spatial working memory in AD mice, revealing a reduced percentage of alternation in 5xFAD mice compared to WT mice. However, this percentage was significantly higher in dCas9-Tet1-injected 5xFAD mice compared to age-matched 5xFAD controls (Figure 4F). Short-term memory was further evaluated using the contextual fear conditioning test, which showed a significant increase in freezing behavior 24 h post-training in dCas9-Tet1-injected 5xFAD mice (Figure 4G). Spatial reference memory and working memory were assessed using the Morris water maze test (Figure 4H-I). Significant differences in escape latency were observed between the control and dCas9-Tet1-injected groups during the visible platform trials (Figure 4H). However, there were no significant differences in the distance traveled between these groups (Figure 4H). In the probe trial, where the platform was removed, dCas9-Tet1-injected 5xFAD mice spent significantly more time in the target quadrant compared to age-matched 5xFAD controls (Figure 4I). We also evaluated the longitudinal impact of dCas9-Tet1 on suppressing AD phenotypes. Notably, consistent with previous results, we observed that the injection of dCas9-Tet1 resulted in a significant decrease in Aβ plaque accumulation and Aβ42 levels (Figure S3A-C), as well as an improvement in spatial working memory (Figure S3D and E). These findings collectively suggest that dCas9-Tet1 administration mitigated cognitive decline and improved memory retention in 5xFAD mice.

## Discussion

Ctsd is a lysosomal aspartate protease responsible for digesting discarded proteins in lysosomes to maintain cellular health, with its expression in various tissues regulated by growth factors and cytokines 55. In the central nervous system, Ctsd is vital for regulating interneuronal communication and neuronal homeostasis 56. Crucially, Ctsd-mediated protein degradation supports neuronal function by breaking down oxidized proteins and unfolded protein aggregates delivered to lysosomes through autophagy or endocytosis 56. Lysosomal pathway abnormalities, which appear early in AD pathology before significant neurofibrillary tangles and plaques accumulate, disrupt this process. Neuronal proteins like huntingtin, alpha-synuclein, and APP can serve as CTSD substrates. If not properly degraded, these proteins may accumulate abnormally and impair neuronal function 55-58. Thus, Ctsd dysfunction is linked to neurodegenerative mechanisms 55-58, and upregulating Ctsd expression could offer potential therapeutic benefits. However, developing effective strategies to increase Ctsd expression remains a challenge.

In this study, our study utilized a dCas9-Tet1-mediated DNA demethylation approach to increase endogenous Ctsd levels in the nervous system, addressing the elevated Aβ burden observed in 5xFAD mice. Prior studies support that Ctsd overexpression in the nervous system can reduce amyloid pathology 58. In the 5xFAD model, baseline Ctsd expression is insufficient to manage the elevated Aβ42 levels 21, 24, 59, and our targeted upregulation approach demonstrates how Ctsd regulation plays a pivotal role in modulating amyloid dynamics. Unlike transient overexpression strategies, such as recombinant protein infusion 60, which may not provide the necessary duration or consistency, our *in vivo* dCas9-Tet1-mediated DNA demethylation approach achieved sustained Ctsd upregulation, with elevated expression levels maintained for up to 144 hours (Figure S2). This extended upregulation appears sufficient to drive effective Aβ42 clearance, particularly in the 5xFAD model, which exhibits high levels of amyloid pathology.

Despite promising results, further research is needed to validate this approach for AD treatment. Since APP degradation involves multiple factors, understanding the key regulatory mechanisms of protein degradation is crucial for targeting Ctsd effectively in AD. Additionally, we observed that dCas9-Tet1-mediated DNA demethylation persisted for up to 8 weeks after the initial injection, but further investigation is necessary to assess the effects beyond 6 months. Confirming the long-term durability of dCas9-Tet1-mediated epigenetic editing is essential for gene therapy applications.

## Conclusion

The dCas9-Tet1 system effectively demethylates the Ctsd promoter region, leading to increased Ctsd expression. This upregulation of Ctsd reduces Aβ42 levels in both *in vitro* and *in vivo* models of AD. Additionally, dCas9-Tet1 treatment improves cognitive function and memory in 5xFAD mice. These results suggest that the feasibility of dCas9-Tet1-mediated Ctsd gene targeting and its therapeutic potential in an AD animal model.

## Supplementary Material

Supplementary figures and tables.

## Abbreviations

Ctsd: cathepsin D
Aβ: amyloid beta
mutAPP: mutant APP
AD: alzheimer's disease
NFTs: plaques and neurofibrillary tangels
pre-proCTSD: pro-procathepsin D
KO: knockout
dCas9: catalytically inactived Cas9
sgRNA: single guide RNA
CGIs: CpG islands
APP: amyloid precursor protein
MEF: mouse embryonic fibroblast
N2a: Neuro-2a
FBS: fetal bovine serum
P/S: penicillin/streptomycin
DMEM: Dulbecco′S Modified Eagle′S Medium
PBS: phosphate-buffered saline
ThT: thioflavin T
DG: dentate gyrus
