# Supplementary material for: Targeted demethylation of cathepsin D via epigenome editing rescues pathology in Alzheimer's disease mouse model
Source: Theranostics. 2025 Jan 1;15(2):428–38. doi: 10.7150/thno.103455 (PMC11671390; doi:10.7150/thno.103455)
Supplement: Supplementary file 1 — Supplementary figures and tables. [file thnov15p0428s1.pdf]

Supplementary Figure 1

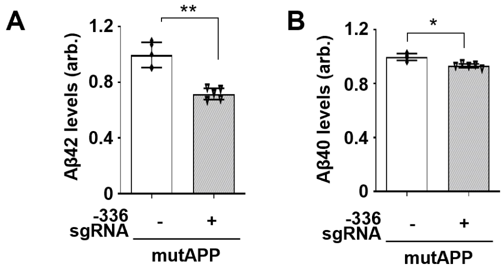

Supplementary Figure 1. Quantification of Aβ42 and Aβ40 levels in mutAPP primary neurons. (A) ELISA analysis of Aβ42 levels in mutAPP mouse primary neurons transduced with −336 sgRNA and dCas9-Tet1. Data are presented as mean ± SEM (n = 5). ***p* < 0.01, two-tailed unpaired t-tests. (B) ELISA analysis of Aβ40 levels in mutAPP mouse primary neurons transduced with −336 sgRNA and dCas9-Tet1. Data are expressed as mean ± SEM (n = 5). **p* < 0.05, two-tailed unpaired t-tests.

Supplementary Figure 2

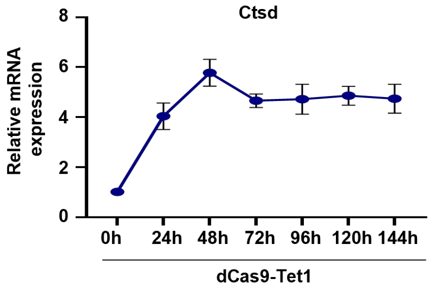

Supplementary Figure 2. Varification of sustained gene activation in mouse brain following dCas9-Tet1 injection. Samples were analyzed at 24, 48, 72, 96, 120, and 144 hours post-injection.

Supplementary Figure 3

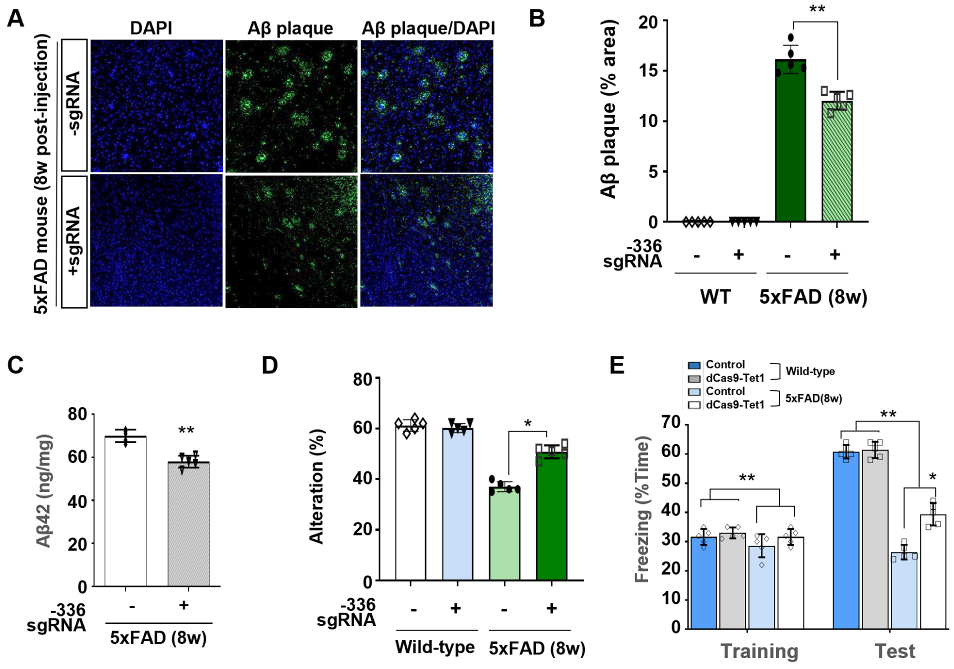

Supplementary Figure 3. Longitudinal effects of dCas9-Tet1-injected 5xFAD mouse model. (A) Immunostaining for ThT in −336 sgRNA and dCas9-Tet1 injected 5xFAD mice on 8 weeks post-injection. (B) Percentage of Aβ plaque in −336 sgRNA and dCas9-Tet1 injected 5xFAD mouse on 8 weeks post-injection. Data are shown as mean ± SEM (n = 5). $^{**}p < 0.01$, two-tailed unpaired t-tests. (C) Levels of Aβ42 measured by ELISA in −336 sgRNA and dCas9-Tet1 injected 5xFAD mice on 8 weeks post-injection. Data are shown as mean ± SEM (n = 5). $^{***}p < 0.001$, two-tailed unpaired t-tests. (D) Y-maze test of −336 sgRNA and dCas9-Tet1 injected 5xFAD on 8 weeks post-injection. Data are shown as mean ± SEM. $^{**}p < 0.01$, two-way ANOVA with Tukey's multiple comparisons test. (E) Fear conditioning test of −336 sgRNA and dCas9-Tet1 injected 5xFAD mice on 8 weeks post-injection. Data are shown as mean ± SEM. $^{**}p < 0.01$, two-way ANOVA with Tukey's multiple comparisons test. Image in panel A is representative of three or more similar experiments.

Supplementary Table 1

Target	Sequence	Chromosome	Position
-336 sgRNA	TATGCAAAAAAGACAGATTAAGG	Chr.7	142387747
-239 sgRNA	ACCTGTATCCAGTCATTCTCCGG	Chr.7	142387758
-151 sgRNA	GCCCGGGCAGTGTTGGAGGCGGG	Chr.7	142387745

Supplementary Table 1. sgRNA sequences targeting Ctsd.

Supplementary Table 2

No.	Target	Chromosome	Position	Direction	Gene
OF1	TATGTCAAAAcAGAaAGATTATGG	Chr8	22288595	-	Tpte
OF2	TATGCAAATAAAcACAGATaAAGG	ChrX	56197821	+	Gm773
OF3	TATGtAAAAAAGACAAGAgTATGG	Chr10	109862984	-	Nav3
OF4	aCATtCAAAAAAGACAGATTAAGG	Chr18	89741195	+	Dok6
OF5	gATGCAAcAAAGACAGACTTAAGG	Chr19	34118103	-	Lipm

Supplementary Table 2. Predicted Ctsd off-target sites for −336 sgRNA. Off-target sites are those with no more than two mismatches compared to on-target sequences. OF, off-target.
